# Effects of COVID-19 on diabetes care among dutch diabetes outpatients

**DOI:** 10.1186/s13098-023-01169-9

**Published:** 2023-10-10

**Authors:** Jessica C.G. Bak, Erik H. Serné, Rolf H.H. Groenwold, Harold W. de Valk, Mark H.H. Kramer, Max Nieuwdorp, Carianne L. Verheugt

**Affiliations:** 1 Department of Vascular Medicine, Amsterdam University Medical Centers, location AMC Meibergdreef 9, Amsterdam, 1105 AZ The Netherlands; 2 Dutch Institute for Clinical Auditing, Leiden, the Netherlands; 3 Leiden University Medical Center, Leiden, the Netherlands; 4 University Medical Center Utrecht, Utrecht, the Netherlands

**Keywords:** COVID-19, Pandemic, Diabetes care, Outpatients

## Abstract

**Aims:**

The COVID-19 pandemic impacted diabetes care by reducing diabetes outpatient visits and diabetes-related screening due to allocation of healthcare resources. Yet the impact of COVID-19 on diabetes outpatients has not been extensively evaluated. This study aimed to assess the effect of the COVID-19 pandemic on diagnostics and intermediate outcomes of outpatient diabetes care pre- and during COVID.

**Methods:**

This observational cohort study included 8,442 diabetes patients in the Dutch Pediatric and Adult Registry of Diabetes (DPARD) visiting diabetes outpatient clinics in 2019 and 2021. A mixed-effects regression analysis was used to examine differences in target achievement of HbA1c, BMI, blood pressure, LDL-cholesterol, eGFR, and the difference in mean HbA1c between 2019 and 2020 among n = 1,426 outpatients who visited in both years. Analyses were adjusted for age, sex, and BMI.

**Results:**

A 22.7% (21.6–23.8%, p < 0.001) decline in outpatient volume was observed during the pandemic (2020). BMI, lipid spectrum, kidney function, and HbA1c were assessed less frequently in 2020 than in 2019. In 2020, compared to 2019, the median HbA1c level increased by 2.2% (1.0 mmol/mol, p = 0.035) and the percentages of patients with known HbA1C meeting targets below 10, 8, 7% (86, 64, and 53 mmol/mol) decreased by 0.5%, 1.7% and 1.4%, respectively. Target blood pressure ≤ 130/80 mmHg was achieved more often in 2020 (15.0% versus 18.3%, p = 0.018), while HbA1c ≤ 86 mmol/mol was achieved less (89.3% versus 87.1%, p = 0.001), among diabetes outpatients seen in both 2019 and 2020. In patients visiting both years, HbA1c was 2.3% (1.9 mmol/l, 95% CI 1.2–2.5, p < 0.001) lower during the pandemic than in the prepandemic (2019).

**Conclusions:**

The COVID pandemic was associated with a marked reduction in patient volume in diabetes outpatient care among five hospitals. Among patients who received outpatient care both before and during the pandemic period, HbA1c control and blood pressure control enhanced during the pandemic. Re-evaluation of current diabetes outpatient care organization is warranted to ensure optimal diabetes care in future times.

**Supplementary Information:**

The online version contains supplementary material available at 10.1186/s13098-023-01169-9.

## Introduction

Since its first report in December 2019, COVID-19 has evolved into a pandemic, with confirmed infections in 400 million people in over 224 countries [1]. The broad clinical spectrum ranges from asymptomatic disease to acute respiratory distress syndrome, systemic complications, and death. Diabetes mellitus is one of the main risk factors for hospitalization and intensive care admission due to COVID-19, and diabetes patients have a 2–3 times higher mortality risk from COVID-19 infection than the general population [2]. Furthermore, the majority of the type 2 diabetes patients suffer from multimorbidity, which in itself is associated with COVID-19 severity [3]. Moreover, overweight and obese COVID-19 patients are at higher risk of developing a severe clinical course than those with a BMI in the normal range, whereas up to 90% of the patients with type 2 diabetes are overweight or obese [4, 5].

Next to the direct effects of COVID-19 infection, the pandemic leads to unprecedented medical, economic, and societal challenges. As COVID-19 overloaded hospital and intensive care units, healthcare resources were allocated from chronic disease management to comply with the increased demand for acute care [6, 7]. Consequently, countries around the globe have been facing challenges regarding the conduct of regular diabetes care and their efforts to adapt care delivery. Furthermore, screening and treatment of complications and cardiovascular risk factors among patients living with diabetes is reduced during the pandemic [8–10]. The COVID-19 era led to additional difficulties for diabetes patients by social distancing, lockdown, working from home, and closing of sports facilities, contributing to a sedentary lifestyle, increased alcohol consumption, and unhealthy eating habits [11–13]. These lifestyle changes and disruption of diabetes care may have implications for diabetes management, glycemic control, and the occurrence of diabetes-related complications. A study from the United States analyzed the effect of the COVID-19 pandemic on diabetes outpatient visits, rates of diabetes-related screening tests, and HbA1c levels. Observed reductions in diabetes outpatient visits and HbA1c testing showed no differences in glycemic control between the pandemic and prepandemic period, [14] however, the study population was limited by certain insurance programs. Whether COVID-19 affects outpatient diabetes care, diagnostics and outcomes on a national level in other Western countries, where patterns of the pandemic spread and coping strategies to preserve adequate diabetes care may have differed, is unknown.

By means of the Dutch nationwide clinical registry DPARD, this study investigates the impact of COVID-19 on various aspects of diabetes outpatient care, on diagnostics and intermediate outcomes including glycemic control, by comparing pre-COVID to during-COVID care.

## Methods

### Study design

This population-based study used data from the Dutch Pediatric and Adult Registry of Diabetes (DPARD). The rationale and design of the DPARD registry has been described in detail previously [15]. In short, DPARD is a nationwide quality registry of adult and pediatric diabetes patients treated in all secondary and tertiary outpatient care across the Netherlands. In the Netherlands, secondary and tertiary outpatient care is provided in hospitals or independent diabetes centers. Five hospitals provided data in 2019 and 2020. Data are collected directly from electronic health records of participating hospitals and entered into batch files, which are data collections. Batch files are uploaded to Medical Research Data Management (MRDM), [16] a trusted third party responsible for securely processing and storing data compliant with all Dutch and European privacy laws [17, 18]. Data are encrypted after entry to prevent data from being traced back to individual patients. Unique non-traceable identification numbers are assigned to every patient to allow for follow-up over time. According to Dutch and European Privacy Protection laws, no ethical approval or informed consent is required for quality research, as DPARD is primarily designed to assess and improve the quality of care. Hospitals are responsible for informing diabetes patients on DPARD participation and the possibility of withdrawing participation.

### Patient selection

In this observational cohort study, we included DPARD patients who received outpatient clinical diabetes care in the Netherlands between January 1, 2019, and January 1, 2021. In secondary and tertiary care all patients are treated with diabetes type 1 and type 2 with inadequate glycemic regulation despite intensive insulin treatment or macroalbuminuria with eGFR < 45 ml/min/1.73m^2^ in patients < 65 years or eGFR < 30 ml/min/1.73m^2^ in patients > 65 years [19]. Outpatient care included both in-person and telemedicine outpatient visits. Telemedicine is any service using electronic information and telecommunication technology to support long-distance clinical healthcare, including video, telephone, internet, and wireless communication [20].

Exclusion criteria are gestational diabetes and diabetes treatment provided by primary care since these patients are not included in DPARD. In the Netherlands, the first wave of the COVID-19 pandemic occurred from March 16 to May 24, 2020, and the second wave from September 21 to December 27, 2020. Therefore, the year 2019 was characterized as the prepandemic period and 2020 as the first year of the COVID-19 pandemic. We subdivided the patients into two cohorts, a 2019 and 2020 cohort, with patients visiting the outpatient clinic before (2019) and during the pandemic (2020). Additionally, we focused on a subgroup of patients who visited the diabetes outpatient clinic in both 2019 and 2020. Diabetes mellitus has been diagnosed according to the guidelines of the American Diabetes Association (ADA) and International Society for Pediatric and Adolescent Diabetes (ISPAD) [21, 22]. Diabetes type was derived from the clinical classification entered in electronic health records by medical professionals. In adult secondary and tertiary diabetes care in the Netherlands, American Diabetes Association (ADA) guidelines [23] are followed, recommending assessment of glycemic status (HbA1C or other glycemic measurement such as time in range or glucose) at least two times a year in patients meeting treatment goals, with a target HbA1c ≤ 53 mmol/mol (≤ 7%). In children treated in Dutch secondary diabetes care, ISPAD (International Society for Pediatric and Adolescent Diabetes) guidelines [24] are used, recommending HbA1c measurement every three months. Kidney function was estimated using the CKD-EPI, MDRD or Cockcroft Gault equation, depending on the equation used in the different hospitals.

### Outcomes

**Fig. 1:**
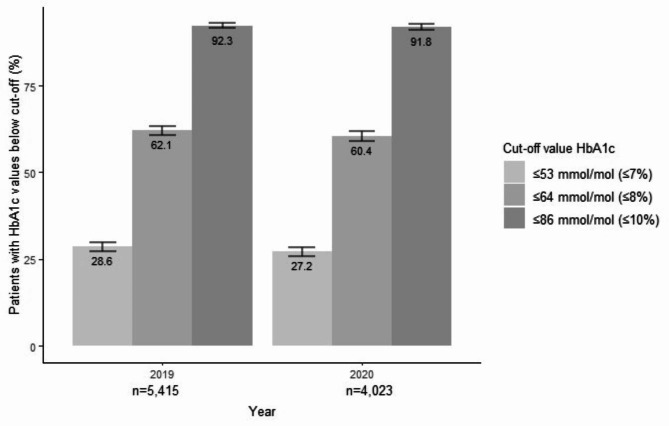
Glucose regulation in DPARD patients receiving diabetes outpatient care in 2019 and 2020. Absolute numbers are expressed as percentages (%). Errorbars depict 95% CI.

**Fig. 2:**
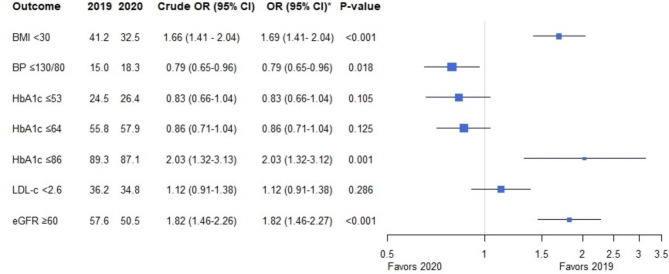
Intermediate outcomes in the subgroup of patients receiving diabetes outpatient care in both 2019 and 2020 N = 1,426 patients who received care in both 2019 and 2020. Numbers are expressed as percentages (%). *Adjusted for sex and age, and body mass index at baseline. 2019 is used as a reference. BMI = Body Mass Index in kg/m^2^, BP = blood pressure in mmHg, HbA1c in mmol/mol, LDL-c = LDL-cholesterol in mmol/l, eGFR in ml/min/1.73m^2^

We defined three subgroups between which overlap partially existed:

1. Patients receiving care in 2019 (n = 5,565; Table 1; Fig. 1).
2. Patients receiving care in 2020 (n = 4,303; Table 1; Fig. 1).
3. Patients visiting in both years 2019 and 2020 (n = 1,426; Table 2; Fig. 2).

**Table 1:** Baseline characteristics of Dutch diabetes outpatients in total and by year of care received

	All patients	Visit in 2019	Visit in 2020
	(n = 8,442)	(n = 5,565)	(n = 4,303)
Age (years)	55.0 (1.0–97.0)	56.0 (4.0–94.0)	55.0 (1.0–97.0)
>50 years (%)	58.0	60.3	57.9
>65 years (%)	27.4	28.4	26.9
>80 years (%)	3.2	3.3	2.9
Children (%)	7.0	5.0	7.3
Adults (%)	93.0	95.0	92.7
Male sex (%)	53.1	52.6	53.1
Diabetes duration (years)	11.0 (0.0–72.0)	12.0 (0.0–71.0)	7.0 (0.0–72.0)
unknown (%)	27.4	34.0	16.1
Smoking status			
smoker (%)	11.6	12.1	11.2
non-smoker (%)	64.5	64.5	61.5
unknown (%)	24.0	23.4	27.3
Diabetes type			
type 1 (%)	26.1	13.2	45.0
type 2 (%)	25.3	22.1	36.4
other/secondary (%)	0.2	0.1	0.4
unspecified (%)	1.9	1.2	3.5
unknown (%)	46.5	63.3	14.7
BMI (kg/m^2^)	27.3 (10.0–49.8)	27.8 (13.6–49.8)	26.8 (10.0–49.7)
<20 (%)	7.0	4.9	7.9
20–24 (%)	20.4	20.1	19.5
25–29 (%)	24.0	24.6	22.8
≥30 (%)	26.6	29.2	23.3
unknown (%)	22.0	21.2	26.4
Cholesterol			
HDL-c (mmol/l)	1.3 (0.2–4.1)	1.3 (0.2–4.1)	1.3 (0.3–4.1)
unknown (%)	16.6	16.0	17.8
LDL-c (mmol/l)	2.4 (0.1–8.4)	2.4 (0.1–7.8)	2.3 (0.1–8.4)
unknown (%)	31.2	32.4	33.5
Blood pressure			
systolic (mmHg)	134.0 (70.0–235.0)	136.0 (87.0–216.0)	132.0 (70.0–235.0)
diastolic (mmHg)	76.0 (40. 0–126.0)	75.0 (40.0–118.0)	77.0 (40.0–126.0)
unknown (%)	75.0	74.1	69.0
Kidney function			
eGFR (ml/min/1.73m^2^)	83.0 (2.0–100.0)	81.0 (2.0–100.0)	83.0 (3.0–100.0)
unknown (%)	26.1	24.9	26.7
albuminuria (mg/l)	10.0 (0.1 - 6175.0)	10.0 (0.1–5135.0)	10.0 (0.2–6175.0)
unknown (%)	28.1	29.2	29.3
HbA1c (mmol/mol)	60.0 (25.0–148.0)	60.0 (25.0–148.0)	61.0 (25.0–148.0)
≤53 mmol/mol (%)	27.5	27.8	25.4
≤64 mmol/mol (%)	59.8	60.4	56.5
≤86 mmol/mol (%)	88.8	89.8	85.9
unknown (%)	3.8	2.7	6.5

Absolute numbers are presented as median (range) or percentages (%). Overlap exists between groups

**Table 2:** Characteristics of patients receiving diabetes outpatient care in both years (n = 1,426)

	2019	2020
Age (years)	58.0 (14.0–90.0)	59.0 (15.0–91.0)
>50 years (%)	65.9	68.0
>65 years (%)	30.1	32.6
>80 years (%)	2.7	3.6
Children (%)	0.2	0.1
Adults (%)	99.8	99.9
Male sex (%)	51.1	51.1
Diabetes duration (years)	3.0 (0.0–71.0)	4.0 (0.0–72.0)
unknown (%)	18.8	16.6
Smoking status		
smoker (%)	12.4	11.8
non-smoker (%)	55.6	57.7
unknown (%)	31.9	30.4
Diabetes type		
type 1 (%)	33.2	33.2
type 2 (%)	46.4	46.4
other/secondary (%)	0.6	0.6
unspecified (%)	3.7	3.7
unknown (%)	16.2	16.2
BMI (kg/m^2^)	28.4 (17.0–49.6)	28.4 (10.0–49.4)
<20 (%)	1.9	3.8
20–24 (%)	16.7	11.8
25–29 (%)	22.6	16.9
≥30 (%)	27.0	22.4
unknown (%)	31.8	45.1
Cholesterol		
HDL-c (mmol/l)	1.3 (0.4–4.1)	1.3 (0.3–4.1)
unknown (%)	18.0	22.7
LDL-c (mmol/l)	2.2 (0.10–6.7)	2.2 (0.1–7.4)
unknown (%)	42.4	45.3
Blood pressure		
systolic (mmHg)	135.0 (96.0–202.0)	134.0 (70.0–235.0)
diastolic (mmHg)	78.0 (44.0–116.0)	78.0 (40.0–126.0)
unknown (%)	53.5	48.2
Kidney function		
eGFR (ml/min/1.73m^2^)	78.0 (5.0–100.0)	77.0 (5.0–100.0)
unknown (%)	23.1	29.1
albuminuria (mg/l)	11.0 (0.3–3557.0)	11.0 (0.6–5555.0)
unknown (%)	35.7	39.6
HbA1c (mmol/mol)	62.0 (27.0–147.0)	61.0 (27.0–148.0)
≤53 mmol/mol (%)	24.5	26.4
≤64 mmol/mol (%)	55.8	57.9
≤86 mmol/mol (%)	89.3	87.1
unknown (%)	1.6	6.4

Absolute numbers are presented as median (range) or percentages (%)

Process parameters BMI (kg/m^2^), blood pressure (mmHg), HbA1c (% and mmol/mol), LDL-cholesterol (mmol/l), eGFR (ml/min/1.73m^2^) and albumin in urine (mg/l) were assessed, as well as the differences in proportion of patients in which these parameters were performed between 2019 and 2020. BMI was calculated as weight in kilograms divided by height squared in meters, using a cut-off value of 25 kg/m^2^ for overweight and 30 kg/m^2^ for obesity. Among patients who visited the outpatient clinic in both 2019 and 2020, differences in HbA1c (% and mmol/mol) were evaluated and the proportion of patients achieving intermediate outcome measures was assessed between both years. Intermediate outcomes were defined as short-term endpoints associated with long-term clinical outcomes [25], including BMI < 30 kg/m^2^, blood pressure ≤ 130/80 mmHg, HbA1c ≤ 53, ≤64, and ≤ 86 mmol/mol (equal to 7, 8 and 10%, respectively), LDL-cholesterol < 2.6 mmol/l, and eGFR ≥ 60 ml/min/1.73m^2^. We estimated the number of patients lost to follow-up due to mortality in our cohort by extrapolating the total death rate per 1,000 inhabitants in the Netherlands from the national cencus (Central bureau of Statistics) to the total of patients lost to follow-up in 2019 and multiplying this by the estimated excess all-cause mortality rate among diabetes patients. The 2019 one-year mortality risk in the general population is 0.008% [26]. In a previous study, excess all-cause mortality was estimated to be up to 4 times higher in the diabetes population than in the general population [27]; therefore, estimated mortality rates were multiplied by 4. Calculated over all patients seen in 2019, the maximum estimated mortality count was 132, which is a maximum of 3.4% of all patients lost to follow-up in 2019.

### Statistical analysis

Descriptive statistics were used to assess patient and disease characteristics. Descriptives of patients receiving care in 2019 or 2020 and both years were provided in tables. In addition, tables with descriptives of adult and pediatric patients were provided. Due to the non-normal distribution of our data,medians and ranges were used for descriptive statistics. Rates of missing values were shown in tables or described in the results. Mixed-effects binary logistic regression models were used to estimate odds ratios (OR) to evaluate differences in target achievement of HbA1c, BMI, blood pressure, LDL-cholesterol, and eGFR between 2019 and 2020 among patients visiting the outpatient clinic in both years. In addition, differences in median HbA1c values between 2019 and 2020 in patients visiting both years were assessed using linear mixed-effects models. The mean HbA1c was log-transformed before applying the mixed model due to the highly skewed data distribution. The results (estimates with 95% confidence intervals) were back-transformed to the original scale using anti-log. HbA1c, BMI, blood pressure, LDL-cholesterol, and eGFR values were clustered within each participant. All models were adjusted for age, BMI > 30 kg/m^2^ at baseline, and sex. Age, sex, BMI > 30 kg/m^2^, and the year of the outpatient visit (2019 or 2020) were treated as fixed effects. The year 2019 was used as the reference year. We used log-transformed values of the non-normally distributed variable age in our analysis to approximate a normal distribution. The models were tested with and without random intercepts to quantify the potential within-subject correlation on the repeated measurements and control for it if necessary. The fits of competing (nested) models were compared using a likelihood-ratio test. P-values below 0.05 were considered statistically significant. Statistical analyses were performed using SPSS (IBM SPSS Statistics for Windows, version 26.0) and R (RStudio, version 1.4.1106).

## Results

In total, 8,442 patients who received diabetes outpatient care in 2019 or 2020 were included, comprising 7,855 adults and 587 children. The median age was 55.0 years (1.0–97.0 years), and 53.1% had male sex. Among patients, diabetes duration was 11.0 years (0.0–72.0 years). Diabetes classification was provided in 53.5% of patients, of whom 26.1% was diagnosed with type 1 diabetes, 25.3% with type 2, and 0.2% with secondary or other causes of diabetes mellitus. Patients were treated across five medical centers (two tertiary and three secondary hospitals), comprising approximately 7% of all Dutch general hospitals. A total of 5,090 patients (60.3%) were followed-up in secondary care and 3,348 (39.7%) in tertiary care.

Table 1 shows the characteristics of all 8,442 patients included in DPARD, as well as patients receiving diabetes outpatient care by year. The number of outpatients decreased by 22.7% (21.6–23.8%, p < 0,001) from 5,565 to 2019 to 4,303 patients in 2020. Diabetes outpatients in 2020 were younger than those in 2019 (55.0 versus 56.0 years, p = 0.004) and had a shorter diabetes duration (7.0 versus 12.0 years, p < 0.001). Compared to 2019, BMI of diabetes outpatients in 2020 was lower (27.8 kg/m^2^ in 2019 versus 26.8 kg/m^2^ in 2020, p < 0.001). Concomitantly, BMI was recorded less frequently in 2020, with performance rates of 78.8% in 2019 and 73.6% in 2020 (p < 0.001). In contrast, blood pressure was measured more frequently in 2020 (31.0 versus 25.9% = p < 0.001), with lower systolic and faintly higher diastolic blood pressure in 2020 compared to 2019 (132/77 versus 136/75 mmHg). Laboratory examinations, including lipid spectrum, kidney function, and HbA1c, were performed slightly less in 2020 than in 2019, with a decline in performance rates ranging from 0.1% in albuminuria to 3.8% in HbA1c.

Diabetes outpatients in 2020 had a 2.2% (1,0 mmol/mol) higher median HbA1c with 7.7% (61.0 mmol/mol) compared to 7.6% (60.0 mmol/mol) in 2019 (p = 0.035). Regarding missing data, the proportion of patients with a known diabetes classification increased from 36.7% to 2019 to 85.5% in 2020, and there were more missing values in diabetes duration in 2019 (34.0%) than in 2020 (16.1%). HbA1c was missing in 150 patients (2.7%) visiting in 2019 and in 280 patients (6.5%, p < 0.001) in 2020. Results of the sensitivity analysis regarding the subgroups of adult and pediatric patients are provided in supplemental Table S1 and S2.

Figure 1 shows the glycemic regulation of patients in DPARD receiving diabetes outpatient care in or 2019 or 2020 with known HbA1c. In addition to the higher HbA1c levels in 2020, the proportion of patients meeting cut-off values below 10, 8, and particularly 7% (86, 64, and 53 mmol/mol) was significantly lower in 2020 compared to 2019 (p < 0.001).

Table 2 and S3 show the 1,426 patients in whom outpatient care was provided in both 2019 and 2020. Compared to patients receiving outpatient care in 2019 or 2020 (n = 8,442), patients with care in both 2019 and 2020 were older (age 59.0–58.0 years). Furthermore, these outpatients had an equal BMI (28.4 kg/m^2^) and worse kidney function (eGFR 77.0–78.0 ml/min and albuminuria 11.0 mg/l). Among patients with outpatient care in both 2019 and 2020, the median BMI remained 28.4 kg/m^2^, while the proportion of patients in whom BMI was assessed declined from 68.2% to 2019 to 55.0% in 2020. Additionally, the percentage of patients < 20 kg/m^2^ increased from 1.9% (95% CI 1.2–2.6) in 2019 to 3.8% (95% CI 2.8–4.8) in 2020, while the percentage of patients ≥ 20 kg/m^2^ decreased compared to 2019. Concomitantly, HbA1c values declined from 62.0 to 2019 to 61.0 in 2020, with an HbA1c-performance rate of 98.4% in 2019 and 93.6% in 2020. Among patients with known HbA1c (in 2019 n = 1,403, 98.4%; in 2020 n = 1,335, 93.6%), targets ≤ 7% (≤ 53 mmol/mol) were achieved in 24.9% (95% CI 22.6–27.1) of patients in 2019 and 28.2% (95% CI 25.8–30.7) in 2020; HbA1c ≤ 8% (≤ 64 mmol/mol) in 56.7% (95% CI 54.1–59.2) in 2019 and 61.9% (95% CI 59.3–64.5) in 2020; and targets of ≤ 10% (≤ 86 mmol/mol) 90.7% (95% CI 89.2–92.2) in 2019 and 93.0% (95% CI 91.7–94.4) in 2020.

Figure 2 shows the target achievement of multiple intermediate outcomes serving as a proxy for long-term clinical outcomes among patients receiving diabetes outpatient care in both 2019 and 2020. Logistic mixed effects modeling was used to assess differences in the achievement of intermediate outcomes BMI < 30 kg/m2, blood pressure ≤ 130/80 mmHg, HbA1c ≤ 7, ≤8, and ≤ 10% (≤ 53, ≤ 64, and ≤ 86 mmol/mol), LDL-cholesterol < 2.6 mmol/l, and eGFR ≥ 60 ml/min between 2019 and 2020 after controlling for sex and age and BMI > 30 kg/m^2^ at baseline. Blood pressure control (≤ 130/80 mmHg) was achieved more often in 2020 compared to 2019 (15.0% versus 18.3%, OR 0.79, 95% CI 0.65–0.96, p = 0.018). A similar yet statistically not significant pattern was observed in the target achievement of HbA1c ≤ 7% (≤ 53 mmol/mol,OR 24.5% versus 26.4%, 0.83, 95%CI 0.66–1.04, p = 0.105) and ≤ 8% (64 mmol/mol, 55.8% versus 57.9%, OR 0.86, 95% CI 0.71–1.04, p = 0.125). In contrast, HbA1c ≤ 10% (≤ 86 mmol/mol) was achieved twice as often in 2019 than in 2020 (89.3% versus 87.1%, OR 2.03, 95% CI 1.32–3.12, p = 0.001), alongside a lower 4.8% performance rate of HbA1c in 2020. In addition, a BMI < 30 kg/m^2^ was found in significantly fewer patients (OR 1.66, 95%CI 1.41–2.04, p < 0.001), coinciding with a 13.3% lower performance of BMI measurements in 2020. Furthermore, eGFR ≥ 60 ml/min was achieved less often as often in 2020 compared to 2019 (OR 1.82, 95% CI 1.46–2.27, p < 0.001).

To evaluate differences in HbA1c values between 2019 and 2020, we used linear mixed modeling on 1,425 patients with known HbA1c visiting the outpatient clinic in both 2019 and 2020. In the unadjusted analysis, mean HbA1c in 2019 was 7.9% (62.8 mmol/mol) and declined by 2.3% (1.8 mmol/mol) from 2019 to 2020 (95% CI 1.2–2.4 mmol/mol, p < 0.001). After adjustment, the mean HbA1c value of 8.2% (66.3 mmol/mol) in 2019 declined with 2.3% (1.9 mmol/mol, 95% CI 1.2–2.5, p < 0.001) to 8% (64.4 mmol/mol) in 2020.

## Discussion

This study showed a decline in diabetes outpatient volume between 2019 and 2020 in five medical centers providing outpatient diabetes care across the Netherlands. In patients visiting in 2019 or 2020 BMI, HbA1c, kidney function, and lipid spectrum were performed less often during the pandemic period in 2020, with a peak decrease in recording of BMI up to 13.3%. Median HbA1c increased from 7.6 to 7.7% (60.0 to 61.0 mmol/mol) in 2020, along with a decrease in the proportion of patients achieving a target-HbA1c below 10, 8 and 7% (86, 64, and 53 mmol/mol). In contrast, mean HbA1c values declined by 2.3% (1.9 mmol/mol), and target blood pressure ≤ 130/80 mmHg were achieved more often in the pandemic compared to the prepandemic period among patients to whom diabetes care was provided in both years. Moreover, targets of BMI < 30 kg/m^2^, HbA1c ≤ 10% (≤ 86 mmol/mol), and eGFR ≥ 60 ml/min were less often achieved during the first pandemic year.

We observed a reduction in diabetes outpatients between the prepandemic and the first year of the COVID pandemic in five medical centers representing approximately 7% of all general hospitals in the Netherlands. Previous studies on the effect of the pandemic on treatment volumes in outpatient care are contradictory. A large cohort study in the United States showed a decrease of 18% in ambulatory contacts of any specialty, both in-person and telemedicine [28]. However, another US study investigating outpatient diabetes care found no clinically relevant difference in outpatient volume between 2019 and 2020, and considerable reductions in outpatient volume in the early pandemic recovered to near-baseline levels at the end of 2020 [14]. In DPARD, such a recovery was not observed. A Dutch population-based cohort studying the impact of COVID-19 on trauma, ICU, cardiovascular, transplantation, oncological, and elective care showed a similar downfall in treatment volumes with a lack of catch-up [29]. Whether the decline in diabetes outpatient volume in 2020 observed in DPARD also appears on a national level is quite likely. The medical centers in this study are situated in the north and the center of the Netherlands. In 2020, the number of confirmed COVID-19 infections among the population differed across the country with an initial peak in the south [30]. Consequently, the effect of COVID on in-hospital care, the need for re-allocation of healthcare, and its impact on outpatient care varied by region. However, over the course of 2020 peaks in COVID-19 infections were observed throughout the country, with an obvious nationwide effect on delivered healthcare [30]. Consequently, the diabetes outpatient volume throughout the Netherlands in all possibility will more or less equally be affected.

DPARD includes patients receiving outpatient care via in-person or telemedicine visits. Although the distinguishment between both types of visits cannot be made by our registry, the decline in the proportion of patients with performed BMI strongly suggests a decrease in in-person visits, which is consistent with literature [28]. DPARD data is directly extracted from electronic health records, and data quality relies on how well data are entered in these records. It is unlikely that diminished data quality would explain the lower performance of BMI, since data quality in DPARD improves every time data is provided by a hospital and our study includes only hospitals delivering data in both 2019 and 2020 [15]. Furthermore, the fact that blood pressure was recorded more frequently in 2020 in patients visiting the outpatient clinic in either 2019 or 2020 seems discordant with a decline in outpatient visits. Yet in patients visiting in both years, blood pressure performance was decreased in 2020, suggesting an increase in data quality over time as a likely explanation. Another possible explanation is that patients were asked to visit the outpatient clinic during pandemic due to worse disease-related characteristics for example blood pressure. Parallel to the reduced number of physical examinations in the pandemic period, a decline in the testing of various laboratory parameters was visible, which might be explained by testing via general practice laboratories or in other hospitals, or merely due to a lower performance coinciding with decreasing outpatient volume. Our findings align with previous studies describing a persistent decrease in volumes of laboratory tests used for monitoring chronic illness [14, 31].

In patients visiting the outpatient clinic in either 2019 or 2020, HbA1c levels increased during the pandemic, while an opposite trend is visible in outpatients receiving care in both years. Of note, the results of patients visiting in either 2019 or 2020 are not corrected for age, BMI and sex since patients visiting in either of these years are two different populations. In contrast, the results of patients seen at the outpatient clinic in both years were corrected. A possible explanation for the in-pandemic Hb1Ac decrease is a lower rate of established diabetes diagnoses during 2020 due to the re-allocation of healthcare resources from chronic disease management and the delay in consultation of healthcare professionals out of fear of getting infected by COVID-19 [32]. This hypothesis is supported by an observed higher admission rate with diabetic ketoacidosis in patients with newly diagnosed diabetes and patients with pre-existing type 2 diabetes in 2020 than in preceding years, suggesting a delay in seeking medical care and worse glycemic regulation [33]. Lifestyle changes and increased stress may also have led to worse glycemic control during the pandemic [34, 35]. Most previous studies evaluating the relation between the pandemic and HbA1c levels did not find changes in glycemic control, [14, 36–38] however, glycemic control in patients receiving care both preceding and during the pandemic has not been studied before. Moreover, governmental measures and restrictions against COVID-19 vary by country and therefore might lead to different social, environmental, and behavioral effects that could impact diabetes mellitus [39]. In our study, patient characteristics of the outpatient population partially varied between 2019 and 2020, thus influencing glycemic control, as outpatients visiting during the pandemic were younger, had shorter diabetes duration, lower blood pressure, and better kidney function than outpatients in the prepandemic. In addition, only 25.6% of the outpatients of 2019 also visited in 2020, indicating a shift in the patient population. Other studies did not show differences in baseline characteristics of patients attending the outpatient clinic during 2019 compared to 2020 [14, 36–38]. Yet there are differences between countries in national hospital capacity and how healthcare services are allocated to avoid exceeding hospital capacity [40]. In the Netherlands, the hospital system is relatively efficient, leading to a sparse overcapacity, negatively impacting the number of patients that can be seen at the outpatient clinic [40, 41]. Differences in patient populations could also explain the opposite effect on HbA1c control found in patients who received care in both years, since they were older, had higher BMI, and had worse kidney function than the total study population. Nevertheless, it has been shown that continuity of care may improve glycemic control, [42] and that support from healthcare professionals encourages self-management resulting in better glycemic control [43].

On-target blood pressure was achieved in significantly more patients during the pandemic than before the pandemic among patients who visited in both years, yet blood pressure recording declined by 5.3% over the same time period. Weight loss probably did not contribute to the observed rise in blood pressure target achievement, since BMI did not change significantly between both years. However, performance of BMI decreased during the pandemic, we cannot establish if differences existed in the proportion of obese patients between both years. Conflicting outcomes have been reported on the impact of the pandemic on blood pressure control, which most likely relies upon the diversity of study populations and study periods [44, 45]. Studies evaluating blood pressure control in the COVID-19 era are focused mainly on home blood pressure monitoring, measurements in general practice, or patients with established hypertension, and mostly did not include diabetes patients or outpatients [44, 45]. An explanation for the improved blood pressure control found in our study may be that hypertension is a risk factor for a worse prognosis after COVID-19 infection, perhaps leading to more conscious behavior concerning lifestyle and management of blood pressure [45]. Another possibility is that patients who visited the outpatient clinic in both years were more concerned about their health, thus displaying behavior that improved blood pressure management leading to better blood pressure control.

The association between the COVID pandemic and effects on Dutch diabetes outpatient care as evaluated in five medical clinics, showed a marked reduction in outpatient volume during the pandemic, with various effects on target achievement between subgroups of outpatients sorted by time span. While our study provides valuable information on the COVID pandemic in hospitals representative of the Dutch healthcare system, DPARD has not reached national coverage yet. However, the number of included hospitals will increase in the following years due to mandatory participation in the registry. In addition, diabetes mellitus is a chronic disease in which the effects of the pandemic on complications, comorbidities, and mortality are expected to show after several years [46, 47]. To gain a complete overview of the effect of the COVID pandemic nationwide data, including long-term follow-up of complications, comorbidity, and survival, is imperative. Moreover, the results found warrant further study into re-evaluation of diabetes outpatient care organization regarding frequency and form of these visits (telemedicine or in-person) in order to identify the optimal format for diabetes target achievement and prevention of complications and comorbidity. The use of various types of glucose monitoring and closed loops should be included in these studies. Such a restructuring may not only give way to further refinement of allocation of healthcare resources, but also makes health care systems more resilient to future COVID peaks or other pandemics.

This study underlines the importance of a national clinical diabetes registry such as ours to gain further insight into diabetes outpatient populations. Nonetheless, our study has limitations. Since only the last outpatient visit and examinations from each year were available, the effect of the COVID pandemic could only be studied over a whole year and not during the COVID peaks themselves. In addition, DPARD data were collected out of the electronic health records and therefore relied on the quality of data registration in these records. Furthermore, one-year follow-up could only be completed in 25.6% of patients, which may have been partially caused by inadequate data delivery out of electronic health records. Since the inclusion of patients relies on declaration data, we do not believe the number of included patients to be lower than the number of patients visiting the outpatient clinic. Moreover, sensor data is not yet included in DPARD but will be included in the future. If time in range was used to monitor glycemic control instead of HbA1c, our study did not include these data. In addition, mortality during follow-up may have directly or indirectly influenced our results, although it is not likely to be significant due to the low estimated mortality rate of 3.4%. Moreover, we did not have information on medication use, some diabetic complications, comorbidities, and their treatment. Finally, data about COVID-19 infection was unavailable, while infection with the virus itself may have impacted HbA1c control.

In conclusion, the COVID pandemic was associated with a marked reduction in patient volume in diabetes outpatient care among five hospitals across the Netherlands. Various clinical and laboratory examinations were performed less during the pandemic. Among patients who received outpatient care in both the prepandemic and pandemic period, HbA1c control and blood pressure control enhanced during the pandemic. Re-evaluation of current diabetes care organization regarding frequency and form of outpatient visits is warranted to identify the optimal format for diabetes target achievement and prevention of complications and comorbidity, in order to ensure excellent diabetes care in future trying times.

### Electronic supplementary material

Below is the link to the electronic supplementary material.

Supplementary Material 1

## Data Availability

The datasets generated and analyzed during the current study are not publicly available, as hospitals delivering data remain ownership of their data. Furthermore, DPARD-data contain information that could compromise research participant privacy but may be available from the corresponding author on reasonable request.
