# Supplementary material for: Effects of COVID-19 on diabetes care among dutch diabetes outpatients
Source: Diabetol Metab Syndr. 2023 Oct 10;15:193. doi: 10.1186/s13098-023-01169-9 (PMC10563332; doi:10.1186/s13098-023-01169-9)
Supplement: Supplementary file 1 — Supplementary Material 1 [file 13098_2023_1169_MOESM1_ESM.docx]

**Supplemental Material.**

Table S1. Baseline characteristics of adult Dutch diabetes outpatients in total and by year of care received

|  | **All patients** | **Visit in 2019** | **Visit in 2020** |
| --- | --- | --- | --- |
|  | (n=7,855) | (n=5,289) | (n=3,398) |
| Age (years) | 57.0 (1.0 - 97.0) | 57.0 (18.0 - 94.0) | 57.0 (18.0 - 97.0) |
| >50 years (%) | 62.4 | 63.4 | 73.3 |
| >65 years (%) | 29.4 | 29.9 | 34.0 |
| >80 years (%) | 3.5 | 3.5 | 3.6 |
| Male sex (%) | 53.1 | 52.6 | 53.1 |
| Diabetes duration (years) | 13.0 (0.0 - 72.0) | 14.0 (0.0 - 71.0) | 8.0 (0.0 - 72.0) |
| unknown (%) | 29.3 | 35.6 | 17.2 |
| Smoking status |  |  |  |
| smoker (%) | 12.3 | 12.6 | 12.0 |
| non-smoker (%) | 65.6 | 64.9 | 63.0 |
| unknown (%) | 22.1 | 22.6 | 25.0 |
| Diabetes type |  |  |  |
| type 1 (%) | 24.3 | 13.9 | 41.3 |
| type 2 (%) | 27.2 | 23.3 | 39.2 |
| other/secondary (%) | 0.2 | 0.1 | 0.4 |
| unspecified (%) | 1.9 | 1.2 | 3.4 |
| unknown (%) | 46.5 | 61.5 | 15.7 |
| BMI (kg/m^2^) | 27.9 (10.0 - 49.8) | 28.3 (15.1 - 49.8) | 27.6 (10.0 - 49.7) |
| <20 (%) | 3.7 | 2.4 | 4.7 |
| 20-24 (%) | 19.2 | 19.3 | 23.6 |
| 25-29 (%) | 25.0 | 25.3 | 22.8 |
| ≥30 (%) | 28.5 | 30.7 | 25.1 |
| unknown (%) | 23.6 | 22.2 | 28.4 |
| Cholesterol |  |  |  |
| HDL-c (mmol/l) | 1.3 (0.2 - 4.1) | 1.3 (0.2 - 4.1) | 1.3 (0.3 - 4.1) |
| unknown (%) | 16.6 | 16.7 | 17.4 |
| LDL-c (mmol/l) | 2.4 (0.1 - 8.4) | 2.4 (0.1 - 7.8) | 2.3 (0.1 - 8.4) |
| unknown (%) | 31.2 | 33.9 | 34.2 |
| Blood pressure |  |  |  |
| systolic (mmHg) | 135.0 (70.0 - 235.0) | 136.0 (87.0 - 216.0) | 133.0 (70.0 - 235.0) |
| diastolic (mmHg) | 76.0 (40. 0 - 126.0) | 75.0 (40.0 - 118.0) | 78.0 (40.0 - 126.0) |
| unknown (%) | 73.9 | 72.8 | 68.1 |
| Kidney function |  |  |  |
| eGFR (ml/min) | 83.0 (2.0 – 100.0) | 81.0 (2.0 - 100.0) | 83.0 (3.0 - 100.0) |
| unknown (%) | 20.6 | 21.0 | 20.9 |
| albuminuria (mg/l) | 10.0 (0.1 - 6175.0) | 10.0 (0.1 - 5135.0) | 10.0 (0.2 - 6175.0) |
| unknown (%) | 29.2 | 30.5 | 29.8 |
| HbA1c (mmol/mol) | 60.0 (25.0 - 148.0) | 60.0 (25.0 - 148.0) | 61.0 (25.0 - 148.0) |
| ≤53 mmol/mol (%) | 24.9 | 25.0 | 22.9 |
| ≤64 mmol/mol (%) | 57.6 | 57.3 | 55.1 |
| ≤86 mmol/mol (%) | 88.8 | 89.1 | 86.4 |
| unknown (%) | 3.4 | 2.8 | 5.8 |

Absolute numbers are presented as median (range) or percentages (%)

Table S2. Baseline characteristics of pediatric Dutch diabetes outpatients in total and by year of care received

|  | **All patients** | **Visit in 2019** | **Visit in 2020** |
| --- | --- | --- | --- |
|  | (n=587) | (n=276) | (n=314) |
| Age (years) | 13.0 (1.0 - 17.0) | 13.0 (4.0 - 17.0) | 14.0 (1.0 - 17.0) |
| Male sex (%) | 53.4 | 55.8 | 51.3 |
| Diabetes duration (years) | 4.0 (0.0 - 16.0) | 4.0 (0.0 - 16.0) | 4.0 (1.0 - 16.0) |
| unknown (%) | 2.0 | 2.5 | 1.9 |
| Smoking status |  |  |  |
| smoker (%) | 1.5 | 2.5 | 0.6 |
| non-smoker (%) | 49.7 | 57.6 | 42.7 |
| unknown (%) | 48.8 | 39.9 | 56.7 |
| Diabetes type |  |  |  |
| type 1 (%) | 50.0 | 0.7 | 93.0 |
| type 2 (%) | 0.0 | 0.0 | 0.0 |
| other/secondary (%) | 0.3 | 0.7 | 0.6 |
| unspecified (%) | 2.6 | 0.0 | 4.8 |
| unknown (%) | 47.1 | 98.6 | 1.6 |
| BMI (kg/m^2^) | 19.9 (13.6 – 43.5) | 19.7 (13.6 – 43.5) | 20.2 (14.0 - 36.3) |
| <20 (%) | 50.7 | 53.6 | 48.4 |
| 20-24 (%) | 36.4 | 35.1 | 37.3 |
| 25-29 (%) | 10.9 | 9.8 | 11.8 |
| ≥30 (%) | 0.7 | 0.7 | 0.7 |
| unknown (%) | 1.0 | 1.3 | 1.3 |
| Cholesterol |  |  |  |
| HDL-c (mmol/l) | 1.6 (0.6 - 3.2) | 1.5 (0.9 - 3.1) | 1.6 (0.6 - 3.2) |
| unknown (%) | 13.3 | 1.8 | 23.6 |
| LDL-c (mmol/l) | 2.3 (0.4 - 6.4) | 2.3 (0.4 - 5.3) | 2.4 (0.6 - 6.4) |
| unknown (%) | 13.4 | 2.5 | 23.6 |
| Blood pressure |  |  |  |
| systolic (mmHg) | 125.5 (81.0 - 136.0) | 125.5 (115.0 - 136.0) | 133.0 (81.0 - 127.0) |
| diastolic (mmHg) | 70.0 (46.0 - 88.0) | 84.0 (80.0 - 88.0) | 70.0 (46.0 - 83.0) |
| unknown (%) | 89.1 | 99.3 | 80.3 |
| Kidney function |  |  |  |
| eGFR (ml/min) | NA | NA | NA |
| unknown (%) | NA | NA | NA |
| albuminuria (mg/l) | 5.0 (2.4 - 1690.0) | 5.0 (2.4 - 292.0) | 5.0 (2.7 - 1690.0) |
| unknown (%) | 13.9 | 4.3 | 22.9 |
| HbA1c (mmol/mol) | 61.0 (32.0 - 136.0) | 59.0 (32.0 - 130.0) | 64.0 (35.0 - 136.0) |
| ≤53 mmol/mol (%) | 19.0 | 26.1 | 13.1 |
| ≤64 mmol/mol (%) | 50.3 | 61.2 | 40.8 |
| ≤86 mmol/mol (%) | 81.8 | 92.0 | 72.6 |
| unknown (%) | 8.3 | 0.0 | 15.9 |

Absolute numbers are presented as median (range) or percentages (%)

Table S3. Baseline characteristics of pediatric Dutch diabetes outpatients by year of care received

|  |  | **2019 or 2020** | **2019 and 2020** |  |
| --- | --- | --- | --- | --- |
|  |  | (n=7,016) | (n=1,426) |  |
| Age (year) |  | 54.0 (1.0-97.0) | 58.0 (14.0-90.0) |  |
| >50 jaar (%) |  | 56.1 | 65.9 |  |
| >65 jaar (%) |  | 26.3 | 30.1 |  |
| >80 jaar (%) |  | 3.1 | 2.7 |  |
| Children (%) |  | 8.3 | 0.2 |  |
| Adults (%) |  | 91.7 | 99.8 |  |
| Male sex (%) |  | 53.5 | 51.1 |  |
| Diabetes duration (years) |  | 13.0 (0.0 - 68.0) | 3.0 (0.0 - 71.0) |  |
| unknown (%) |  | 29.6 | 18.8 |  |
| Smoking status |  |  |  |  |
| smoker (%) |  | 11.5 | 12.4 |  |
| non-smoker (%) |  | 65.8 | 55.6 |  |
| unknown (%) |  | 22.6 | 31.9 |  |
| Diabetes type |  |  |  |  |
| type 1 (%) |  | 24.6 | 33.2 |  |
| type 2 (%) |  | 21 | 46.4 |  |
| other/secondary (%) |  | 0.1 | 0.6 |  |
| unspecified (%) |  | 1.6 | 3.7 |  |
| unknown (%) |  | 52.7 | 16.2 |  |
| BMI (kg/m^2^) |  | 27.1 (10.0 - 49.8) | 28.4 (17.0 - 49.6) |  |
| <20 (%) |  | 7.6 | 1.9 |  |
| 20-24 (%) |  | 22.2 | 16.7 |  |
| 25-29 (%) |  | 25.4 | 22.6 |  |
| ≥30 (%) |  | 27.4 | 27 |  |
| unknown (%) |  | 17.4 | 31.8 |  |
| Cholesterol |  |  |  |  |
| HDL-c (mmol/l) |  | 1.3 (0.2 - 3.9) | 1.3 (0.4 - 4.1) |  |
| unknown (%) |  | 15.3 | 18.0 |  |
| LDL-c (mmol/l) |  | 2.4 (0.10 - 8.4) | 2.2 (0.10 - 6.7) |  |
| unknown (%) |  | 28.4 | 42.4 |  |
| Blood pressure |  |  |  |  |
| systolic (mmHg) |  | 134.0 (81.0 - 216.0) | 135.0 (96.0 - 202.0) |  |
| diastolic (mmHg) |  | 74.0 (40.0 - 118.0) | 78.0 (44.0 - 116.0) |  |
| unknown (%) |  | 80.4 | 53.5 |  |
| Kidney function |  |  |  |  |
| eGFR (ml/min) |  | 84.0 (2.0 - 100.0) | 78.0 (5.0 - 100.0) |  |
| unknown (%) |  | 25.5 | 23.1 |  |
| albuminuria (mg/l) |  | 10.0 (0.3 - 6175.0) | 10.0 (0.3 - 3557.0) |  |
| unknown (%) |  | 25.8 | 35.7 |  |
| HbA1c (mmol/mol) |  | 60.0 (25.0 - 148.0) | 62.0 (27.0 - 147.0) |  |
| ≤53 mmol/mol (%) |  | 24.4 | 24.5 |  |
| ≤64 mmol/mol (%) |  | 56.7 | 55.8 |  |
| ≤86 mmol/mol (%) |  | 87.5 | 89.3 |  |
| unknown (%) |  | 4.5 | 1.6 |  |

Absolute numbers are presented as median (range) or percentages (%)
